# Twelve-Month Outcomes in Patients with Obesity Following Bariatric Surgery—A Single Centre Experience

**DOI:** 10.3390/nu15051134

**Published:** 2023-02-23

**Authors:** Radu Petru Soroceanu, Daniel Vasile Timofte, Madalina Maxim, Razvan Liviu Platon, Vlad Vlasceanu, Bogdan Mihnea Ciuntu, Alin Constantin Pinzariu, Andreea Clim, Andreea Soroceanu, Ioana Silistraru, Doina Azoicai

**Affiliations:** 1 Department of Surgery I, “Grigore T. Popa” University of Medicine and Pharmacy, 700115 Iasi, Romania; 2 3rd Surgical Unit, Department of Surgery, “St. Spiridon” County Clinical Emergency Hospital, 700111 Iasi, Romania; 3 2nd Surgical Unit, Department of Surgery, “St. Spiridon” County Clinical Emergency Hospital, 700111 Iasi, Romania; 4 Department of Morpho-Functional Sciences II, “Grigore T. Popa” University of Medicine and Pharmacy, 700115 Iasi, Romania; 5 “Socola” Institute of Psychiatry, 700282 Iasi, Romania; 6 Department of Social Work, Journalism, Public Relations and Sociology, Faculty of Social Sciences and Humanities, Lucian Blaga University, 550024 Sibiu, Romania; 7 Department of Preventive Medicine and Interdisciplinarity, “Grigore T. Popa” University of Medicine and Pharmacy, 700115 Iasi, Romania

**Keywords:** obesity, bariatric surgery, diabetes, weight loss, body composition, T2DM, LSG, RYGB

## Abstract

Background: As obesity rates continue to rise worldwide, many surgeons consider bariatric procedures as a possible cure for the upcoming obesity pandemic. Excess weight represents a risk factor for multiple metabolic disorders, especially for type 2 diabetes mellitus (T2DM). There is a strong correlation between the two pathologies. The aim of this study is to highlight the safety and short-term results of laparoscopic sleeve gastrectomy (LSG), Roux-en-Y gastric bypass (RYGB, laparoscopic gastric plication (LGP) and intragastric balloon (IGB) as methods used in the treatment of obesity. We followed the remission or amelioration of comorbidities, tracked metabolic parameters, weight loss curves and hoped to outline the profile of the obese patient in Romania. Methods: The target population of this study was represented by patients (n = 488) with severe obesity who qualified for the metabolic surgery criteria. Starting from 2013 to 2019, patients underwent four types of bariatric procedures and were subsequently monitored over the course of 12 months in the 3rd Surgical Clinic at “Sf. Spiridon” Emergency Hospital Iași. Descriptive evaluation indicators, as well as those of analytical evaluation were used as statistical processing methods. Results: A significant decrease in body weight was recorded during monitoring and was more pronounced for patients who underwent LSG and RYGB. T2DM was identified in 24.6% of patients. Partial remission of T2DM was present in 25.3% of cases, and total remission was identified in 61.4% of patients. Mean blood glucose levels, triglycerides, LDL and total cholesterol levels decreased significantly during monitoring. Vitamin D increased significantly regardless of the type of surgery performed, while mean levels of vitamin B12 decreased significantly during monitoring. Post-operative intraperitoneal bleeding occurred in 6 cases (1.22%) and a reintervention for haemostasis was required. Conclusions: All procedures performed were safe and effective methods of weight loss and improved associated comorbidities and metabolic parameters.

## 1. Introduction

As the prevalence of obesity nearly tripled during the last three decades, it has become a global public health issue. Obesity is associated with considerable morbidity and mortality rates and could reverse the current trend of increasing life expectancy [1]. Prevention efforts are difficult due to the multifactorial nature of the disease and treatment is always challenging. Obesity is a result of the complex interaction between human behaviour, environmental factors and genetic predisposition. It represents the most important risk factor for multiple chronic diseases, such as T2DM, cardiovascular, pulmonary, renal and oncological pathologies [2,3,4]. The World Health Organization reported more than 1.9 billion overweight and 650 million obese adults in 2016 [5]. It already represents a burden to the economy with massive healthcare costs. Therefore, obesity prevention and treatment demand the attention of researchers and the scientific community [6]. Obesity is classified with the aid of the body mass index (BMI).

Available treatment for obesity includes lifestyle changes through diet and physical exercise, medication and invasive procedures known as bariatric or metabolic surgery. Numerous studies report that the surgical approach has superior results compared to non-surgical controls [7,8,9].

To date, bariatric surgery is the most effective treatment both in terms of weight loss and weight loss maintenance. Literature data also show amelioration or remission of weight associated comorbidities [2,3,4]. Bariatric surgery is considered complex due to the anatomical and physiological changes that develop in the gastrointestinal tract. The morbid profile of the obese patient is also a challenge. Technical advances, especially the introduction of laparoscopy, have substantially reduced the risks associated with these procedures [10]. 

Although bariatric surgery is very efficient, there is still controversy regarding what the optimal type of procedure is. For patients with severe clinical obesity and high anaesthetic risk, Douglas Hess described biliopancreatic diversion with duodenal switch (BPD-DS). This procedure was developed as an improvement to BPD. By preserving the pylorus, Hess has eliminated some common shortcomings such as dumping syndrome, biliary reflux and anastomotic ulcerations [11]. The surgery was performed in two steps in order to minimize perioperative risk related to technical complexity and long operative time. A longitudinal sleeve gastrectomy (LSG) that removed over 80% of the stomach was initially performed. Satisfied by the results, both in weight loss and comorbidity improvement, many of the patients never came back and LSG became a standalone procedure. It has become one of the most performed procedures worldwide due to reduced technical difficulty and optimal results. It is a purely restrictive procedure and can be easily converted to Roux-en-Y gastric bypass (RYGB) if results are inadequate or complications arise [12]. LSG is not recommended in patients with gastroesophageal reflux disease (GERD) or hiatal hernias (HH) because it can worsen their symptoms.

RYGB was initially described in the 1970s based on observations regarding unintentional weight loss in patients with peptic ulcer disease who underwent gastric resections. It combines the principles of restriction and malabsorption and is, to this day, one of the most efficient procedures both in terms of weight loss and remission rates of associated comorbidities [13].

Laparoscopic gastric plication was introduced in 2007 as a more conservative, reversible and cheaper alternative to LSG. It is a restrictive procedure in which a double plication of the greater curvature of the stomach is performed. It is preferred by patients because it does not alter the physiology of the stomach and does not require a resection. However, long-term results are debatable [14].

Endoscopic treatment is the least invasive option. The intragastric balloon (IGB) is a temporary restrictive procedure that occupies part of the stomach and induces early satiety. Aimed to fill the gap between surgery and medication, it is the preferred method for patients with low grade obesity or for those reluctant to undergo more invasive procedures [15].

Depending on the BMI and associated comorbidities, the surgeon together with the patient will choose the most appropriate procedure. 

Consulting a multidisciplinary team in a specialized centre before surgery can help to manage the patient’s modifiable risk factors, reduce perioperative complications and optimize outcomes. 

## 2. Materials and Methods

The target population of this study consisted of patients with obesity who failed to lose weight through diet and physical exercise. They underwent four types of bariatric surgical procedures from 2013 to 2019 and were subsequently monitored over the course of 12 months in “St. Spiridon” Emergency Clinical Hospital Iași, 3rd Surgical Clinic.

The subject selection was performed by non-random sampling and data collection was performed retrospectively, longitudinally, based on the information contained in the medical documents of each patient. 

**Figure 1:**
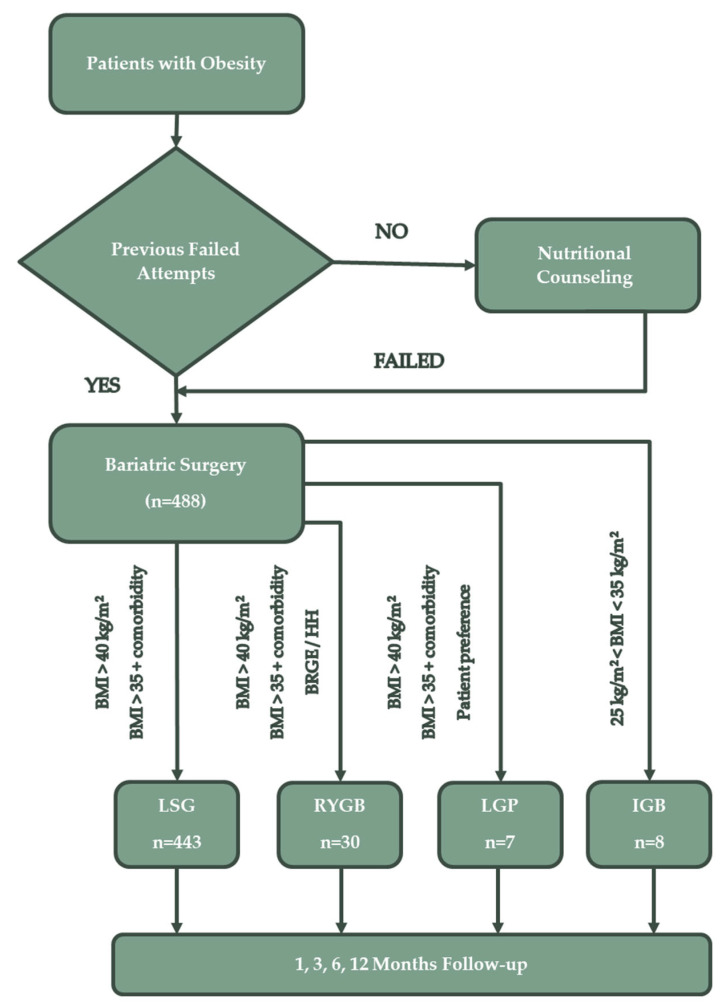
The selection and inclusion process for bariatric surgery candidates.

We included 488 patients who underwent bariatric surgery during the study period. They were followed up at 1, 3, 6 and 12 months after the procedure. LSG was performed on 443 patients, RYGB on 30 patients, 7 patients opted for LGP and 8 patients for IGB (Figure 1).

At the time of the study, eligibility conditions for bariatric procedures were in accordance with the 1991 National Institutes of Health (NIH) Consensus Statement recommendations, as follows [16]:

- patients with a BMI > 40 kg/m^2^ with or without coexisting medical conditions and who do not present a high anaesthetic-surgical risk;
- patients with a BMI > 34.9 kg/m^2^ with one or more obesity-related comorbidities or with a significant impairment in quality of life (T2DM, essential hypertension, dyslipidaemia, sleep apnoea syndrome or non-alcoholic fatty liver disease—NAFLD);
- patients over 18 years old.

The procedures performed were LSG (n = 443), RYGB (n = 30), LGP (n = 7) and IGB (n = 8).

During the study period, we monitored the main demographic and morphological characteristics, the evolution of comorbidities associated with obesity and the biological parameters in each of the surgical groups.

The data were extracted from medical charts, then uploaded and processed using the statistical functions in SPSS 18.0. Due to the longitudinal data collection, all patient data protection provisions were enforced, as the medical team in the teaching hospital were well informed on data and patients’ rights protection.

In calculating the differences between two or more groups at the 95% significance threshold, depending on the distribution of the value series, the t-Student test, the F test (ANOVA), the χ^2^ test, the Pearson correlation coefficient (r) and the ROC curve were applied to the quantitative variables.

## 3. Results

### 3.1. Patient Distribution

**Figure 2:**
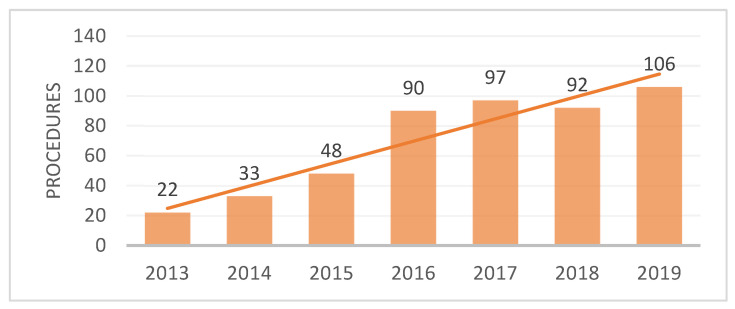
Number of bariatric procedures performed annually.

In our centre, the first LSG procedures were performed in 2013. Since then, the number of procedures performed annually has followed an increasing trend (Figure 2). Most of the interventions were performed in 2019 (n = 106), until all elective surgeries were prohibited at the beginning of 2020 due to the global COVID-19 pandemic. 

### 3.2. Demographic Characteristics

In the study group, 63.9% of the patients were female, with a sex ratio of F/M = 1.8/1.

The age of the patients ranged from 18 to 70 years, with a mean of 40.81 years, close to the median of 41 years, suggesting a normal distribution of the series of values. The highest frequency was registered in the fourth decade of life (31.4%).

### 3.3. Distribution by Type of Procedure Performed

The most common procedure was LSG, with a proportion of 90.8% (443 patients). RYGB represented 6.14% of the interventions (30 patients), 1.4% were LGP (7 patients) and 1.6% were IGB (8 patients). The average post-operative length of stay was 2.79 days.

### 3.4. Preoperative Comorbidities and Morphologic Characteristics

Patient weights ranged from 77 to 216 kg, with an overall average of 132.33 ± 23.07 kg.

Preoperative BMI ranged from 29.11 to 66.48 kg/m^2^, with a mean of 45.69 ± 10.81 kg/m^2^ in patients treated with RYGB and 45.01 ± 6.84 kg/m^2^ in patients treated with LSG. These values were significantly higher than those recorded in patients treated with LGP (35.20 ± 1.92 kg/m^2^) or IGB (32.13 ± 1.70 kg/m^2^) (*p* = 0.001).

Essential hypertension was identified in 30.53% of patients. Of them, 27.66% of patients had stage I hypertension, 1.84% had stage II and 1.02% had stage III. All patients were under chronic antihypertensive medication.

Dyslipidaemia was present in 48.77% of patients, hepatomegaly in 66.8% of cases and NAFLD in 81.76% of patients.

Vitamin D deficiency was identified in 53.07% of the cases, and 30 patients (6.14%) presented gastroesophageal reflux disease (GERD) symptoms.

T2DM was identified in 1/4 of the patients in the study group (24.6%), with slightly higher frequencies in the group of patients who underwent IGB (37.5%) and in the patients who underwent LSG (25.3%). The differences were not statistically significant (*p* = 0.313). 

Sleep apnoea syndrome (SAS) was identified in 88.1% of the study group, with 69.9% of patients having a severe form. It occurred more frequently in women (62.6% vs. 37.4%; *p* = 0.078) in the over 40 age group (46% vs. 54%; *p* = 0.571) and in those with urban residence (61.6% vs. 38.4%; *p* = 0.564).

### 3.5. Outcomes

**Figure 3:**
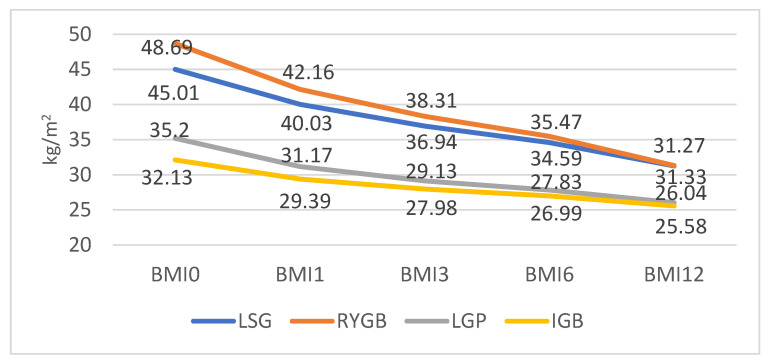
Mean BMI during the 1, 3, 6 and 12-month follow-ups.

Regardless of the type of procedure performed and the age and gender of the patient, the mean BMI has decreased from one follow-up to another during the 12 months (*p* < 0.001): from 45.01 ± 6.84 kg/m^2^ to 31.27 ± 4.46 kg/m^2^ for LSG, from 48.69 ± 10.81 kg/m^2^ to 31.33 ± 4.63 kg/m^2^ for RYGB, from 35.20 ± 1.92 kg/m^2^ to 26.04 ± 3.18 kg/m^2^ for LGP and from 32.13 ± 1.70 kg/m^2^ to 25.58 ± 1.30 kg/m^2^ for IGB (Figure 3).

**Figure 4:**
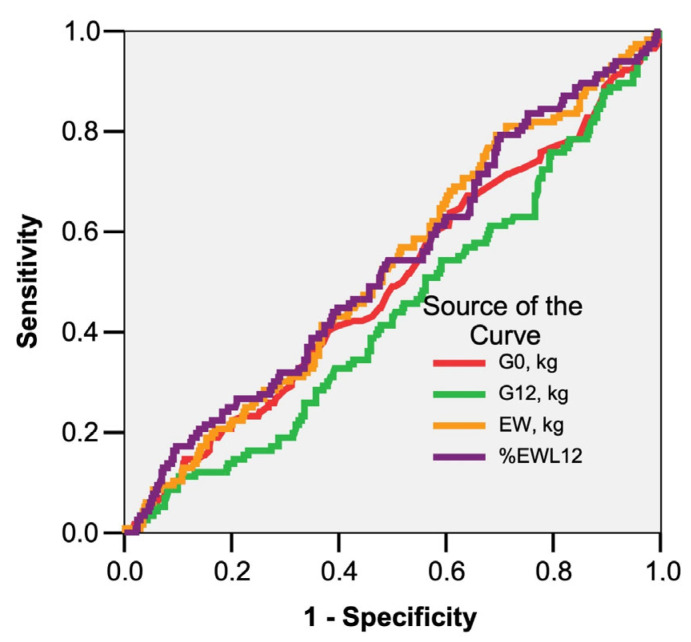
Initial weight and excess weight as predictors of T2DM total remission.

Partial remission of T2DM was present in 25.3% of the study group and total remission was identified in 61.4% of patients. Plotting the ROC curve, it is highlighted that neither the preoperative weight (G0-AUC = 0.493; 95% CI: 0.433–0.553; *p* = 0.819) nor the excess weight (EW-AUC = 0.527; 95% CI: 0.469–0.586; *p* = 0.369) were good predictors for partial or total remission of T2DM (Figure 4). Fasting glucose levels at 12 months in the presence or absence of glucose-lowering pharmacologic treatment were used to define total or partial remission.

The mean preoperative weight was significantly higher in patients with apnoea than in those without apnoea (133.55 vs. 123.31 kg; *p* = 0.001). Lower follow-up weight loss is also identified throughout the monitoring. One year after the procedure, the average weight remained significantly higher in patients with apnoea syndrome (92.41 vs. 86.45 kg; *p* = 0.003), but the percentage of weight lost at 12 months (%EWL12) did not differ significantly compared to patients without apnoea syndrome (*p* > 0.05).

**Figure 5:**
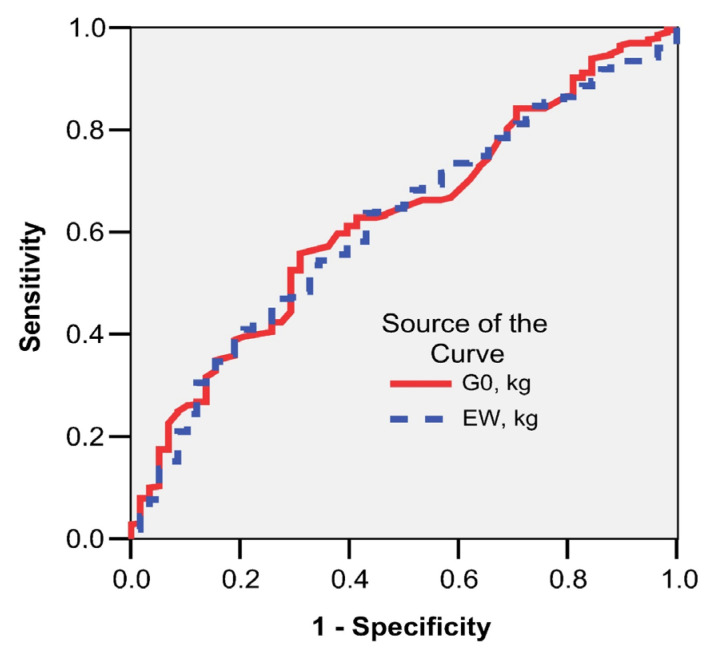
Initial weight and excess weight as predictors for sleep apnoea syndrome.

Plotting the ROC curve highlights that both the preoperative weight (G0-AUC = 0.623; 95% CI: 0.551–0.696; *p* = 0.002) and the excess weight (EW-AUC = 0.615; 95% CI: 0.542–0.687; *p* = 0.004) were good predictors of the presence of sleep apnoea syndrome (Figure 5).

Total cholesterol and LDL cholesterol levels decreased significantly during monitoring, from an average of 248.51 ± 46.04 mg/dL to 193.43 ± 41.11 mg/dL (*p* = 0.001) and from 227.85 ± 77.63 mg/dL to 97.42 ± 17.52 mg/dL, respectively (*p* = 0.001), and did not correlate with any specific type of intervention.

Triglyceride levels decreased significantly during monitoring, from a mean value of 350.40 ± 160.77 mg/dL to 212.81 ± 136.73 mg/dL (*p* = 0.001) at the end of the follow-up.

Mean blood glucose levels decreased significantly during monitoring, from an average of 112.53 ± 60.88 mg/dL to 91.38 ± 15.77 mg/dL (*p* = 0.001).

Vitamin D was dosed before surgery and at 3, 6 and 12 months. During monitoring, values increased significantly regardless of the type of surgery applied from 21.43 ± 11.36 ng/dL to 35.31 ± 11.39 ng/dL (*p* < 0.001). At the same time, the mean vitamin B12 levels decreased significantly during monitoring, from an average of 497.60 ± 215.83 pg/dL to 463.81 ± 211.41 pg/dL (*p* = 0.001). All monitored metabolic parameters were centralised in the adjacent table (Table 1).

We consider it necessary to define the parameters used to describe weight loss:

- Body Mass Index (BMI) = Weight (kg)/Height^2^ (m);
- Ideal Body Weight (IBW) = 50 + [0.91 × (height in cm − 152.4)] in men;
- Ideal Body Weight (IBW) = 45.5 + [0.91 × (height in cm − 152.4)] in women;
- Excess Weight (EW) = Actual weight − IBW;
- Percentage of Weight Loss (%EWL) = (postoperative weight loss)/(preoperative EW) × 100.

## 4. Discussion

The aim of the study is to report clinically relevant data and to highlight the results of bariatric surgical procedures regarding weight loss and the subsequent effect on ameliorating comorbidities and metabolic parameters. The validation of our results by comparison is challenging, as populations have different social, economic, and cultural backgrounds and eating habits. We thus hope to outline the metabolic profile and morphological characteristics of the patient with obesity in a middle-income Eastern European country.

The first bariatric procedures were performed in 2013 and were exclusively LSG for the first two years. This procedure was also performed the most during the span of the study (90.8%). The number of procedures performed increased annually. LSG is currently the most practiced procedure worldwide. This is due to the safety profile of the intervention, with a steeper learning curve and lower rates of intraoperative and postoperative complications. Comparable efficiency and results as RYGB are proven in numerous studies [17,18,19]. As the experience of the surgical team grew, more complex bariatric procedures were performed. In addition, patients became more aware that obesity is a serious health problem and started asking for a surgical solution. LSG does not require gastro-intestinal anastomoses and there are fewer short- and long-term complications associated with the procedure. 

Initially described by E. Mason in 1967, perfected and standardized by Griffin et al. in 1977, RYGB remains the cornerstone of metabolic surgery to this day, combining both the principles of restriction and malabsorption. RYGB was gradually introduced in our centre and performed more often alongside some redo procedures, surgical management of complications (migrated adjustable gastric bands) and 14 Single Anastomosis Duodeno-Ileal Switch (SADIS), but these patients were not included in the current study.

Analysing the demographic characteristics of the patients in this study, especially gender distribution, we can observe that 63.9% of patients were female, with a sex ratio of F/M = 1.8/1. Other studies also confirm a higher prevalence of obesity among the female population: 42.48 vs. 33.85% [20,21].

One cross-sectional study carried out on a cohort of 3361 patients follows the association between BMI and depressive disorder. It emphasizes a U-shaped relationship between the two pathologies. They tend to be more strongly associated in underweight male patients and in female patients with a higher BMI. This may be due to the social promotion of a more muscular body type among men, while a leaner body type is more desirable among females. This may contribute to a higher addressability to bariatric surgery for females [22].

A closer analysis pertaining to the age of our patients highlights that almost 60% are between the ages of 30 and 49, a socio-economically active population, thus emphasizing the importance of finding the optimal solution.

The mean weight at the time of surgery is higher among patients who underwent RYGB. This is in line with the literature data, as this type of intervention is especially indicated for patients with higher levels of obesity and those for whom LSG is not recommended. Contraindications for LSG include addictive behaviour towards sweet foods with a high caloric index, symptomatic GERD or hiatal hernias. In these cases, LSG would aggravate the symptoms [23,24]. In addition, while LSG is exclusively a restrictive procedure, RYGB also adds malabsorption to the equation. This is achieved by altering the anatomy of the digestive tract, shortening the path that food has to travel and implicitly lowering the amount of nutrients that are being absorbed [25].

Although the long-term results are comparable between LSG and RYGB, the associated comorbidities and the particularities of each patient must be considered. Regardless of the type of surgery chosen, the best results will be obtained by patients who understand the risks, benefits and assume responsibility for their diet and regular post-operative clinical and biological follow-ups [26].

The most important parameters that describe the dynamics of the weight curve are represented by BMI and %EWL. In this study, there were no significant differences gender-wise. The BMI values had a favourable trend at all timepoints, regardless of the type of intervention performed. This uniform variation can be attributed to the fact that patients with LGP and IGB had lower mean weight values at the time of surgery than those with LSG and RYGB and associated fewer comorbidities. Similar results are reported in some long-term studies [27,28].

The role of bariatric surgery in the partial or total remission of T2DM is already well established and some guidelines even recommend extending surgical indications to patients with class I obesity in whom glycaemic control under optimal drug treatment is inadequate [2,7]. In our study group, T2DM was identified in 24.6% of patients, with a slightly higher frequency in the LSG group. For these patients, the mean preoperative weight was similar to that of non-diabetic patients, but %EWL12 was slightly higher in the T2DM group. Plotting the ROC curve highlighted that neither preoperative weight nor excess weight were good predictors of partial or total remission of T2DM. Remission criteria were defined as follows [29]:

- complete T2DM remission—fasting plasma glucose < 100 mg/dL and/or HbA1c < 6% for at least 1 year after surgery in the absence of glucose-lowering pharmacologic treatment;
- partial T2DM remission—fasting plasma glucose < 126 mg/dL and/or HbA1c < 6.5% without antidiabetic medication for at least 1 year.

Complete remission spanning over 5 years or more is considered curative [29]. A randomized clinical trial reports a remission rate of 66.7% at two years after surgery, similar to our investigation [30]. However, some patients relapsed during the 10-year monitoring period, but glycaemic control remained satisfactory. It seems that one important factor leading to remission is the hypocaloric state induced by the prolonged caloric deficit following bariatric procedures. Some studies on patients with T2DM highlighted the immediate improvement of insulin sensitivity similar to their surgical counterparts just by having a caloric restriction, similar to that in the first 10 to 20 days after bariatric surgery [31]. This effect was mainly attributed to the amelioration of the hepatic insulin sensitivity. The favourable effects of insulin on skeletal muscles were observed later on and were more weight loss dependent. Incretin and insulin levels are both severely altered in obese patients with T2DM. However, they seem to return to normal values shortly after bariatric surgery, especially in those who underwent RYGB [32]. The factors and action mechanisms leading to remission are still unclear. It seems that patients with a more substantial weight loss have higher chances than others. In addition, the duration of T2DM prior to surgery, poor glycaemic control and intensive use of insulin seem to negatively influence remission rates. These patients also have a higher chance of relapse [33,34]. Lack of remission should not be considered as a failure of bariatric surgery. Long term control of all metabolic comorbidities is equally as important and the International Diabetes Federation even advocates for the complementary use of medication to prolong and enhance the effects of surgical procedures [34]. As of November 2022, the American Society of Metabolic and Bariatric Surgery (ASMBS) and the International Federation for the Surgery of Obesity and Metabolic Disorders (IFSO) updated the criteria for bariatric surgical procedures as follows:

- patients with a BMI > 35 kg/m^2^ with or without coexisting medical conditions and who do not present a high anaesthetic-surgical risk;
- patients with a BMI of 30–34.9 kg/m^2^ with one or more obesity-related comorbidities or with a significant impairment in quality of life (T2DM, essential hypertension, dyslipidaemia, sleep apnoea syndrome or NAFLD).

Due to numerous studies that demonstrate the efficacy, long-term results and safety, bariatric surgery may now be considered for adolescents and patients over 70 years that are appropriately selected. These indications are widely accepted and the evidence indicates superior results compared to non-surgical interventions in terms of both weight loss and glycaemic control [35].

All interventions had a favourable outcome on total and LDL cholesterol at all time-points. The lipid profile improved or normalized during the 12-month period of monitoring.

In our study group, obstructive sleep apnoea (OSA) was the main factor that delayed the surgical procedure. As seen in the data presented above, severe forms were diagnosed in patients unaware of this pathology. They required continuous positive airway pressure (CPAP) treatment and re-evaluation from the pneumonologist in order to assess the improvement in the apnoea–hypopnea index (AHI). Poor oxygenation may be responsible for ischaemia in sutured, resected or anastomosed tissue which is a predisposing factor to anastomotic leaks. Undiagnosed patients present higher risks of postoperative cardiac events and respiratory failure [36]. These are the main reasons bariatric candidates require preoperative screening, treatment, postoperative monitoring and extensive follow-ups. Polysomnography is the gold standard for diagnosing OSA [36]. Although few studies focus on sleep apnoea improvement in patients who underwent bariatric procedures, they demonstrate the superior outcomes in the surgical group compared to the common medical care group [37,38]. At the end of the monitoring period, some patients in the control group had an AHI increased by five and a higher BMI than the baseline values. In contrast, RYGB had a significant impact on OSA remission or improvement in moderate and severe forms [38]. Regarding our patients, the ROC curve highlights that both the preoperative and the excess weight were good predictors of the presence of OSA. The obstructive aetiology of apnoea syndrome may explain this result. In patients with severe obesity, excess fat in the cervical extremity may cause compression of the upper airways when in the supine position.

A randomized, double-blind clinical study among patients with obesity, altered basal blood glucose and hypovitaminosis D observed that correcting vitamin D deficiency improves insulin resistance and reduces the risk of progression to T2DM [39]. Furthermore, our patients’ serum levels of vitamin D show a significant increase during monitoring due to the initiation of nutritional supplementation aimed to correct hypovitaminosis and protein deficiency starting with the first month postoperatively. 

The role of vitamin D in modulating the immune system has been proven in numerous studies. The low vitamin D levels among the European general population represent a public health problem. They have been associated with a predisposition to infections and chronic diseases [40]. A recent study concludes that individuals with low vitamin D levels are 80% more likely to acquire a COVID-19 infection compared to a control group with normal levels. Moreover, obesity and overweight are positively associated with higher rates of mortality in SARS-CoV-2 infections. A strong association is also underlined in previous MERS and SARS epidemics [41]. Vitamin D can be obtained from exogenous sources (nutrition or supplementation) or synthetized in the presence of UV-B light, but the levels vary depending on season and latitude of residence [42]. Some studies report that the effects of vitamin D deficiency could be reversible. Supplementation proved a favourable outcome increasing both the size and number of aged skeletal muscle fibres. It also improved muscle strength and balance in laboratory animals [43]. Among several murine animal models, some studies mention the beneficial effects of prolonged vitamin D administration on adipose tissue remodelling. The histological findings suggest that it can regulate adiposity, decrease lipid accumulation and prevent sarcopenia [44,45].

Micronutrient deficiencies are common after bariatric surgery. Therefore, many patients need routine vitamin and mineral supplementation. These changes can also be seen in our study, especially in the case of vitamin B12 found only in exogenous sources. These deficiencies could be explained by reduced dietary intake and anatomical and physiological changes in the gastrointestinal tract, especially in the case of malabsorptive procedures. However, the incidence of vitamin and mineral deficiencies following LSG compared to RYGB have yet to be reported [46]. At the end of the study, the levels of vitamin B12 decreased significantly, thus confirming the necessity of long-term supplementation with iron and vitamin B12 in addition to multivitamins and general minerals. Unmonitored patients may develop anaemia caused by altered gastrointestinal absorption.

Short term complications consisted of six post-operative intraperitoneal bleedings in patients with LSG. Laparoscopic reintervention during post-operative day 1 for definitive haemostasis was required in all cases. Bleeding occurred either from the trocar site, divided gastro-colic ligament or at the level of the remaining stomach due to imperfect stapling. Pertaining long-term complications, we could mention a case of small bowel volvulus caused by an entero-parietal flange and a case of common bile duct obstruction due to migrated gall stones. Both patients had underwent RYGB and the latter was treated through a laparoscopic gastrotomy in the excluded part of the stomach to allow access to the duodenum and to endoscopically remove the gall stones. This was caused by the anatomical changes in the GI tract that occur as a consequence of RYGB. 

Limitations of the study include incomplete data availability. Although the patient pool was higher, some were not compliant with the follow-up program and could not be included in the study. Some patients had their residency abroad or in other regions. Long term results are not reported. Although our recommendation for post-bariatric follow-up is every 6 months after the first year, few patients presented long-term. A more procedure-oriented comparison could not be performed as the vast majority of our patients underwent LSG. 

Strengths include the large sample size, the close monitoring of the main metabolic parameters throughout the first 12 months, and outlining the profile of the patient with obesity in a developing Eastern European country. This study is also among the first reports of bariatric surgery outcomes in Romania. We hope to continue following our patients further in order to gain a better understanding of their needs and expectations.

## 5. Conclusions

At 12 months after surgery, the weight loss percentage was not significantly correlated with a lower or higher preoperative weight.

All bariatric procedures were effective methods of weight loss and improved weight associated comorbidities and laboratory parameters. The outcomes of LSG and RYGB were comparable and both procedures had better outcomes in terms of weight loss than LGP and IGB.

Choosing the right type of bariatric surgery should take into account each patient’s comorbidities, weight loss goals and the individual anaesthetic-surgical risk.

Situations where patients did not have associated weight-related comorbidities were very rare. Patients were usually underdiagnosed, and some pathologies were diagnosed during preoperative investigations, sometimes postponing the surgical procedure. Most frequently, patients associated high blood pressure, dyslipidaemia, hepatomegaly, NAFLD, vitamin D deficiency, OSA and T2DM. 

After more than half a century of research, an ideal solution is yet to be found. Obesity is a progressive chronic disease with complex and incompletely elucidated mechanisms. Complete treatment cannot be achieved by restrictive or malabsorptive surgery alone. The best results can be achieved by having an informed patient, a well-trained surgeon and a multidisciplinary team.

## Figures and Tables

**Table 1:** Metabolic parameters variation during follow-ups.

		Baseline	1 Month	3 Months	6 Months	12 Months
	LSG	45.01 ± 6.84	40.03 ± 6.01	36.94 ± 5.46	34.59 ± 5.04	31.27 ± 4.46
BMI	RYGB	48.69 ± 10.81	42.16 ± 8.16	38.31 ± 6.65	35.47 ± 5.71	31.33 ± 4.63
kg/m^2^	LGP	35.20 ± 1.92	31.17 ± 2.93	29.13 ± 2.94	27.83 ± 2.96	26.04 ± 3.18
	IGB	32.13 ± 1.70	29.39 ± 1.55	27.98 ± 1.48	26.99 ± 1.37	25.58 ± 1.30
	LSG	113.28 ± 63.54	95.3 ± 14.87	94 ± 12.08	88.92 ± 8.7	91.3 ± 15.49
Gluc.	RYGB	105.16 ± 18.47	94.02 ± 24.9	91.37 ± 7.17	88.3 ± 13.51	89.35 ± 17.68
mg/dL	LGP	102.12 ± 28.2	99.73 ± 19.7	97.8 ± 15.46	94.7 ± 9.31	96.49 ± 17.91
	IGB	100.61 ± 20.06	98.41 ± 16.53	98.29 ± 14.1	87.61 ± 17.22	93.93 ± 21.71
	LSG	247.97 ± 46.74	237.49 ± 46.1	221.91 ± 43.76	207.33 ± 42.74	193.33 ± 41.51
Chol.	RYGB	256.93 ± 39.53	245.87 ± 39.99	228.9 ± 39.95	212 ± 39.82	196.13 ± 39.66
mg/dL	LGP	236.86 ± 39.79	228.43 ± 37.56	212.14 ± 37.11	197.29 ± 35.79	184.14 ± 34.04
	IGB	257.25 ± 33.41	244.88 ± 33.5	228.63 ± 33.34	217.88 ± 37.05	197.13 ± 33.43
	LSG	226.53 ± 77.63	-	171.67 ± 47.11	138.48 ± 28.47	97.15 ± 17.38
LDL	RYGB	244.63 ± 85.71	-	175.57 ± 45.32	138.30 ± 25.68	100.5 ± 18.59
mg/dL	LGP	223.29 ± 80.8	-	195 ± 45.69	146 ± 19.06	102.86 ± 17.28
	IGB	242.25 ± 87.6	-	197.5 ± 38.72	132.75 ± 31.72	95.75 ± 23.06
	LSG	43.84 ± 14.89	-	57.58 ± 15.49	66.59 ± 15.68	74.15 ± 15.84
HDL	RYGB	46.33 ± 13.25	-	60.60 ± 14.35	69.50 ± 14.66	77.23 ± 15.27
mg/dL	LGP	45.29 ± 12.09	-	60.86 ± 10.81	69.43 ± 11.31	78 ± 10.74
	IGB	39.50 ± 18.07	-	51.38 ± 17.82	61.63 ± 17.16	69.88 ± 17.37
	LSG	350.11 ± 161.2	296.33 ± 155.7	258.18 ± 150.3	233.31 ± 143.4	211.18 ± 137.4
Triglyc.	RYGB	371.43 ± 151.9	321.17 ± 137.4	284.1 ± 137.21	263.6 ± 130.91	242.5 ± 125.86
mg/dL	LGP	180.86 ± 107.1	154 ± 89.95	127.29 ± 79.95	106.29 ± 73.9	104 ± 65.66
	IGB	436.38 ± 111.3	356.13 ± 128.1	323.38 ± 132.6	300.88 ± 134.4	286.88 ± 127.9
	LSG	19.11 ± 9.33	-	21.41 ± 11.38	29.54 ± 11.44	35.47 ± 11.42
Vit D	RYGB	18.9 ± 13.21	-	21.13 ± 11.87	29.40 ± 11.87	35.28 ± 11.87
ng/dL	LGP	20.35 ± 11.27	-	24.68 ± 12.67	32.95 ± 12.67	38.83 ± 12.67
	IGB	20.55 ± 15.84	-	21.22 ± 8.2	29.49 ± 8.2	35.37 ± 9.45
	LSG	501.20 ± 217.1	501.72 ± 215.6	488.42 ± 214.2	479.03 ± 212.7	468.37 ± 211.8
Vit B12	RYGB	440.67 ± 167.9	432.8 ± 181.05	419.80 ± 181.1	408.77 ± 181.1	397.67 ± 181.2
pg/dL	LGP	461.57 ± 292.3	477.43 ± 274.1	463.00 ± 274.7	451.29 ± 274.9	438.14 ± 275.8
	IGB	543.25 ± 232.1	518.88 ± 228.4	501.00 ± 235.3	497.25 ± 225.3	481.50 ± 231.7

## Data Availability

The data presented in this study are available on request from the corresponding author.
